# Metagenomic cross-talk: the regulatory interplay between immunogenomics and the microbiome

**DOI:** 10.1186/s13073-015-0249-9

**Published:** 2015-11-20

**Authors:** Maayan Levy, Christoph A. Thaiss, Eran Elinav

**Affiliations:** Immunology Department, Weizmann Institute of Science, 100 Herzl Street, Rehovot, 76100 Israel

## Abstract

The human microbiome, often referred to as the ‘second genome’, encompasses up to 100-fold more genes than the host genome. In contrast to the human genome, the microbial genome is flexible and amenable to change during the host’s lifetime. As the composition of the microbial metagenome has been associated with the development of human disease, the mechanisms controlling the composition and function of the metagenome are of considerable interest and therapeutic potential. In the past few years, studies have revealed how the host immune system is involved in determining the microbial metagenome, and, in turn, how the microbiota regulates gene expression in the immune system. This species-specific bidirectional interaction is required for homeostatic health, whereas aberrations in the tightly controlled regulatory circuits that link the host immunogenome and the microbial metagenome drive susceptibility to common human diseases. Here, we summarize some of the major principles orchestrating this cross-talk between microbial and host genomes, with a special focus on the interaction between the intestinal immune system and the gut microbiome. Understanding the reciprocal genetic and epigenetic control between host and microbiota will be an important step towards the development of novel therapies against microbiome-driven diseases.

## The bi-directional interaction between microbial and host genomes

The genomic revolution has transformed our ability to study the composition and evolution of genomes. These technical advances have enabled comprehensive analysis of the human genome and of the genomes of all indigenous commensal microorganisms, collectively referred to as the microbial metagenome. The human microbiome is acquired after birth and is shaped throughout an individual’s lifetime. The individual's diet [1], household characteristics [2], exposure to xenobiotics [3], and the time of day [4] are among the environmental factors that most strongly influence the metagenome.

The microbiota reaches a stable configuration at age 2–3 years (Table 1). The species and strain composition of the microbiota are generally maintained over the following years, while undergoing fluctuations according to the environmental factors mentioned above. The community structure and relative abundance of members of the microbiota differ markedly among individuals [5, 6]. The human microbiota is dominated by bacteria belonging to two phyla, *Bacteroidetes* and *Firmicutes* [7]. A core set of more than 50 distinct bacterial species is found in nearly all human individuals [8]. A corresponding functional core likewise exists and is encoded by the minimal human gut metagenome, which is required for the correct function of the intestinal ecosystem. The core functional features of the minimal human gut metagenome include functions important for proper interaction between the host and its microbiome, such as degradation of complex polysaccharides and synthesis of short-chain fatty acids [8].

**Table 1:** Postnatal development of the microbiota and effects of obesity and IBD

Physiologic or pathologic status	Involved taxa	Phenotypic manifestation	References
Early microbial colonization	Breastfed individuals: low species diversity, most abundant phyla are *Actinobacteria* and *Firmicutes*. Formula-fed individuals: low species diversity, most abundant phyla are *Actinobacteria* and *Bacteriodetes*. All individuals: facultative anaerobic bacteria including *Staphylococcus* species, *Streptococcus* species, *Escherichia coli* and other *Enterobacteriaceae* are thought to be the first colonizers of the gut	Several factors can control microbial colonization after birth, including delivery mode, hygiene, feeding, and antibiotics use	[122, 123]
	*Weissella*, *Leuconostoc*, *Staphylococcus*, *Streptococcus*, and *Lactococcus* species predominate	Human colostrum and breast milk contains more than 700 species of bacteria, including species typically found in the oral cavity	[124]
	Major phyla: *Bacteriodetes* and *Firmicutes*	From 18 months, the diet influences the bacterial composition, and this composition is stabilized, with increased diversity	[125]
	*Enterococcaceae*, *Streptococcaceae*, *Lactobacillaceae*, *Clostridiaceae*, and *Bifidobacteriaceae*	The newborn gut microbiota is similar to the maternal skin and vaginal microbiota	[123]
	Cesarean‐born infants featured lower relative abundance of *Bifidobacterium* and *Bacteroides* species compared to vaginally born children	The delivery mode determines early colonization through modulating the type of bacteria that the child is exposed to at birth	[126, 127]
Obesity	Reduced abundance of *Bacteroidetes* species in obese individuals	Transfer of microbiota with a composition characteristically found in obese mice into germ-free mice results in weight gain	[12]
	*Firmicutes* were dominant in normal-weight individuals and in those with obesity, but substantially less abundant in individuals who had undergone gastric bypass surgery, who had a proportional increase in abundance of *Gammaproteobacteria. Prevotellaceae* were highly enriched in individuals with obesity	Treatment with antibiotics results in weight gain	[128]
	Increased relative abundance of *Faecalibacterium prausnitzii* in children with obesity	Treatment of children with antibiotics before the age of 6 months results in weight gain	[129]
	*Bacteroides* and *Clostridium* were the most prevalent genera	Colonization of adult germ-free mice with a microbial community harvested from the distal gut of conventionally raised mice produces a dramatic increase in body fat content within 10–14 days	[130]
	The relative abundance of *Bacteroidetes* in obese mice is 50 % lower than that in normal-weight mice, whereas the relative abundance of *Firmicutes* is proportionally higher	A low-calorie, fat-restricted or carbohydrate-restricted diet leads to an increase in relative abundance of *Bacteroidetes*	[13]
IBD	Higher relative abundance in CD: *Proteobacteria*, *Fusobacterium*, *Haemophilus influenzae* and other *Haemophilus* species, *Neisseriaceae*, *E. coli*. Lower relative abundance in CD: *Bacteroides*, *Bifidobacterium*, and *Blautia*	The intestinal microbiota is disturbed in children with IBD	[115, 131]

*CD* Crohn’s disease, *IBD* inflammatory bowel disease, *UC* ulcerative colitis

The composition of the gut metagenome is highly relevant for human health and disease, as illustrated by findings pertaining to the etiology of obesity (Table 1). Human energy homeostasis is determined by a set of known human genes, yet these genes account for only a small amount of the variability in energy homeostasis that exists between humans. In addition to the human genes, the gut microbiota has been suggested to affect energy balance by influencing the efficiency of calorie harvest from the diet [9, 10]. Obesity is characterized by lower bacterial diversity, along with alterations in the bacterial metagenome [11, 12]. Similarly, the development of obesity in leptin-deficient mice (ob/ob) coincides with phylum-level changes in the gut microbiome, with obese mice having reduced relative abundance of *Bacteroidetes* and increased relative abundance of *Firmicutes* [13].

Advances in genomics have also led to a revolution in the study of another factor that shapes the microbiome: the host immune system. Basic and clinical immunology have been redefined by developments in next-generation sequencing and genome editing techniques [14]. Our molecular understanding in this field now ranges from transcriptome profiles of innate and adaptive immune cells to epigenetic regulation of cytokine expression and the effect of genetic mutations on immune-mediated diseases.

Combining the techniques of metagenomics and immunogenomics has led to an understanding of how the microbiome influences the development and activity of the immune system, and is in turn itself shaped by the various arms of the host immune system [15]. Studies in mouse models have revealed that impairment in this genomic cross-talk is closely associated with the pathogenesis of numerous multi-factorial diseases, which include infectious diseases, inflammatory and autoimmune disorders, metabolic diseases, cancer, and even neurodegenerative and aging-related disorders [16]. As such, advances in genomic research have important implications for human disease and are rapidly expanding the horizons of immunology towards fields and disorders never studied before, which is expected to result in the identification of innovative and personalized genomic-based immune-mediated and microbiome-mediated therapeutic strategies. To facilitate this development, a basic understanding of the mechanisms by which host and microbial genomes influence one another is critically important. In the following sections, we review some of the common principles by which the microbiota influences the host genome and by which host immunogenomics, in turn, control the microbiome. We also point out the largest gaps that remain in our knowledge of the mechanisms underlying this metagenomic cross-talk and discuss future avenues in this very young field of study.

## Genomic control of the host by the microbiome

The microbiota has emerged as a central factor that influences host gene expression at all body sites that feature commensal colonization, such as the skin, respiratory tract, gastrointestinal tract, and the urogenital system. Generally, the microbiota can exert its gene regulatory influence either directly through the exposure of the host to live microorganisms or components of microbial cells [17], or indirectly through the secretion of metabolites [18]. However, whether an observed effect is due to microbial antigens or metabolites has not been elucidated in the majority of cases. Below, we first highlight the general principles whereby the microbiota influences host gene expression at different body sites, with a focus on the gastrointestinal tract. We then concentrate on the mechanisms of gene regulation in cells of the immune system and discuss examples of how the microbiota influences immune cell differentiation and function at the levels of transcription, DNA methylation, and histone modification.

### The microbiome as a regulator of host gene expression

**Fig. 1:**
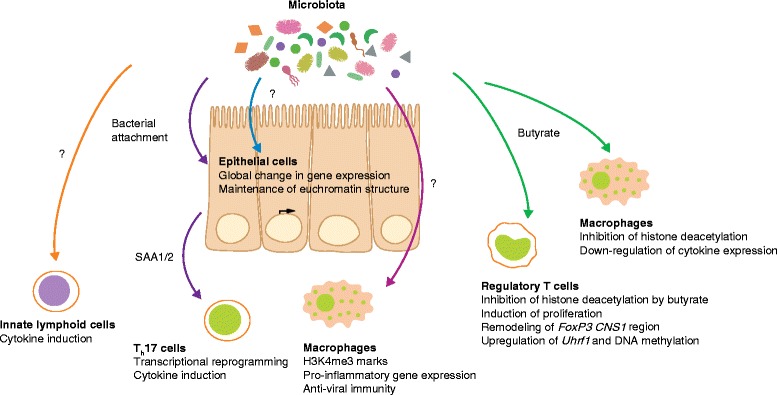
Effects of the microbiota on host gene regulation. Examples of epigenetic and transcriptional changes induced by commensal colonization in the intestine. *Arrows* denote bacterial signals involved in the genetic reprogramming of immune cell subsets and intestinal epithelial cells. *Question marks* indicate unknown microbial signals involved in the regulation of gene expression in macrophages, innate lymphoid cells, and intestinal epithelial cells. *T*_*h*_ *17* T helper 17

The notion that the activity of commensal microorganisms may influence genomic organization and gene regulation of the mammalian host gained momentum about a decade ago, when studies of intestinal tissue using DNA microarrays found major changes in gene expression between germ-free mice and mice colonized with members of the commensal microbiota [19, 20]. Colonization by a single commensal bacterium modifies gene expression in a large variety of physiological processes, ranging from nutrient metabolism and tissue development to immune system function and antimicrobial activity [19]. Similarly, even a single enteric virus, upon introduction into a germ-free setting, dramatically shapes the global gene expression profile of the intestine [21]. When entire microbial communities are introduced into germ-free mice, transcriptional responses to microbial colonization can be observed all along the gastrointestinal tract [22] and are specific for different cellular compartments [23]. Similarly, upon microbial colonization after birth, intestinal gene expression undergoes dramatic reprogramming, which is partially dependent on microbial sensing receptors of the innate immune system [22, 24]. This observation suggests that successive stages of commensal colonization after birth are involved in shaping gene regulatory processes during intestinal maturation (Fig. 1). These studies support the notion of a 'super-organism' in which eukaryotic and prokaryotic genomes cross-regulate each other [25]. Of note, the transcriptional response to microbial colonization is in part species-specific, as, for instance, gene regulation differs between mice and zebrafish that have undergone reciprocal microbiota transplantation [26].

Despite the enormous influence of intestinal microbial colonization on gene regulation processes in the intestine and in other systems [27–29] the mechanisms mediating this transcriptional reprogramming remain largely elusive. The first insights on these mechanisms came from studies focusing on selected examples of the microbial effects on the host epigenome [30]. The methylation levels of the gene encoding Toll-like receptor 4 (*Tlr4*) were found to be lower in germ-free mice, which indicates that commensal bacteria may exert their gene regulatory effect through epigenetic modifications [31]. Furthermore, mice with a conditional deletion of the histone deacetylase 3 (*Hdac3*) gene in intestinal epithelial cells developed aberrations in intestinal barrier function, loss of Paneth cells, and enhanced susceptibility to intestinal inflammation [32]. These aberrations depend on microbiota-derived signals, as germ-free mice lacking intestinal Hdac3 do not have the same phenotype. When the microbiota of Hdac3-deficient mice was transferred to Hdac3-sufficient germ-free mice, intestinal inflammation was not observed, which indicates that an intact microbiota-Hdac3 axis is required for intestinal barrier maintenance.

Seminal work by Camp et al. [33] indicated that the modulatory effect of the microbiota on intestinal gene expression happens independently of the spatial organization of nucleosome-depleted accessible chromatin (Fig. 1). Germ-free mice had a chromatin accessibility landscape similar to that of conventionally raised mice or germ-free mice into which normal commensal microbes had been introduced. This finding suggests that the effects of commensal bacteria on intestinal gene regulation occur through distinct signaling events that influence transcription factor binding to open chromatin. Identifying these signaling events may unravel the pathways utilized by the host tissue to assess the microbial colonization status and appropriately respond by modifying its transcriptome.

Together, these first studies analyzing the regulatory mechanisms underlying the genomic cross-talk between microbiome and host genome indicate that epigenetic modification of gene expression through the microbiome might involve multiple non-redundant pathways that integrate microbial signals into appropriate host transcriptional responses. Deciphering the mechanisms underlying microbiome-regulated gene expression could complement advances in our understanding of non-genomic processes by which the microbiota influences epithelial function, including glycosylation [34] and cargo sorting [35], which have been reported in the past couple of years.

### Microbiome-mediated gene regulation in the immune system

The influence of the microbiota on genetic and epigenetic regulation of gene expression in the host is especially apparent in the immune system, both in homeostatic development and function of the system and in disease. In a human study that correlated the mucosal microbiome composition with host gene expression in the pelvic pouch of patients with inflammatory bowel disease (IBD), the strongest associations between microbes and host gene expression were found in pathways of the innate and adaptive immune system, most prominently in cytokine pathways and the complement system [36]. This finding points toward a critical role for the immune system in microbiome-mediated genomic regulation of the host. Indeed, upon microbial colonization of germ-free mice, major transcriptional induction of innate and adaptive immune genes occurs as early as 4 days after microbial inoculation, and includes activation of expression of antimicrobial peptides, lineage transcription factors of T cells, cytokines, and molecules involved in antigen presentation [37]. Microbial colonization thus has a major role in the regulation of gene expression in immune cells [17], and the first mechanisms by which commensal bacteria regulate immunogenomics have begun to be unraveled [18, 38, 39]. Whereas many reviews have highlighted the role of the microbiota in shaping immune system development and function [16], here we focus specifically on the gene regulatory aspects of the microbiota in the immune system (Table 2).

**Table 2:** Examples of reprogramming of the immunogenome by the microbiota

Cell type	Influence	Microbial signal(s)	References
Macrophages	Deposition of activating histone marks, enhanced cytokine expression	Unknown	[40, 132]
Macrophages	HDAC inhibition, reduced cytokine expression	Butyrate	[41]
Regulatory T cells	HDAC inhibition, acetylation of *FoxP3* CNS1 region, induction of proliferation, upregulation of *Uhrf1*	Butyrate	[42–45]
T helper 17 cells	Transcriptional reprogramming through epithelially produced SAA1 and SAA2	Epithelial attachment	[47, 48]
Neutrophils	Induction of neutrophil aging, steady-state granulopoiesis, stimulation of migration through SAA1 and SAA2	TLR ligands	[133–135]
Innate lymphoid cells	Transcriptional reprogramming, cytokine induction	Unknown	[133–135]
Natural killer T cells	Mucosal recruitment via CXCL16, cytokine production	Glycosphingolipids	[52, 136, 137]
γδ T cells	Transcriptional reprogramming	Unknown	[51]

*HDAC* histone deacetylase, *TLR* Toll-like receptor

Myeloid cells of the intestinal mucosa are among the first line of innate host defense against invading bacteria and thus require rapid transcriptional responses against changes in microbial colonization. Examples of such regulation include the induction of expression of pro-inflammatory genes, such as genes encoding type I interferons, in intestinal mononuclear phagocytes upon microbial colonization [40]. These effects are mediated at least in part through the deposition of trimethylation marks on histone H3 at the transcriptional start sites of these genes, which renders them transcriptionally more active [40] (Fig. 1). Conversely, the microbial short-chain fatty acid butyrate downregulates pro-inflammatory gene expression in intestinal macrophages through inhibition of histone deacetylation [41].

Short-chain fatty acids also mediate transcriptional control in regulatory T cells [42–44]. Butyrate increases the number of peripheral regulatory T cells through inhibition of histone deacetylation in intronic enhancer sequences of the *FoxP3* locus [43, 44], which encodes the lineage transcription factor of regulatory T cells. Consequently, treatment with butyrate ameliorated T-cell-dependent colitis development in mice. In addition to histone acetylation, DNA methylation also seems to have a role in regulatory T-cell homeostasis in response to intestinal microbial colonization. The microbiota induces the expression of the gene encoding the DNA methylation adaptor protein UHRF1 (*Uhrf1*) [45]. The expression of this protein is necessary for the maintenance of proper DNA methylation in colonic regulatory T cells, as well as for their proliferation and function (Fig. 1).

Another T-cell subset strongly influenced by the microbiota is the T helper 17 (T_h_17) lineage [46]. Epithelial attachment of intestinal bacteria drives transcriptional reprogramming of intestinal epithelial cells, which in turn secrete mediators such as serum amyloid A1 and serum amyloid A2 (which are encoded by *Saa1* and *Saa2*, respectively) to regulate gene expression in T_h_17 cells resident in the lamina propria [47, 48]. Although the exact mechanisms underlying this transcriptional reprogramming remain to be fully understood, this finding demonstrates that the microbiota programs expression of host immune system genes not only through secreted metabolites, but also through biogeographical localization and attachment.

One of the most prominent yet poorly understood examples of microbial regulation of host immune cell gene expression is found in innate lymphoid cells (ILCs). ILCs are found at mucosal surfaces of the body and respond to microbial challenges by producing cytokines. According to the type of cytokines produced, one can distinguish between ILCs type 1, 2, and 3. Type 3 ILCs respond to intestinal microbial colonization through the production of interleukin (IL)-22 [49] and ILCs from germ-free mice or antibiotic-treated mice fail to produce this cytokine, which is important for intestinal barrier function and host defense against enteric pathogens [50]. The microbial signals that mediate gene expression in ILCs, including those that induce IL-22 expression, remain unknown (Fig. 1).

Another instance of close cross-talk between the microbiota and immune cells has been found in mucosal γδ T cells and natural killer T (NKT) cells. These cells, albeit expressing a T-cell receptor, have some characteristics of innate immune cells, such as rapid cytokine secretion in response to conserved classes of stimuli. The microbiota reprograms the transcriptome of intraepithelial γδ T cells, including reprogramming of the expression of antimicrobial peptides [51]. Furthermore, commensal microbiota colonization during the neonatal period in mice decreased CpG methylation in the 5' region of the gene encoding the chemokine CXCL16 [52]. The resultant decreased levels of *Cxcl16* expression protected the mice from enhanced mucosal accumulation of NKT cells in the lung and gastrointestinal tract. As a consequence of ameliorated NKT cell recruitment, bowel inflammation and allergic asthma were mitigated by neonatal colonization [52].

These examples indicate that the regulatory effect of the microbiota on host immunogenomics and epigenomics is a pleiotropic phenomenon with considerable influence on inflammatory processes in health and disease (Table 2). Although most studies examining the influence of the microbiome on immune system function have focused on the gastrointestinal tract, we should also note that other body surfaces feature similar phenomena, including the skin [53] and lung [54]. Further understanding of the mechanisms by which commensal bacteria and their products orchestrate immune cell gene expression will be instrumental to move this young field of study forward to eventually harness the new insights for therapeutic purposes.

## Genomic control of the microbiome by the host

Owing to the enormous effect of the microbiome on host gene expression and the resultant effect on human health, identifying the mechanisms by which bacterial communities are assembled and structured is of substantial interest [55, 56]. As discussed above, two main factors are responsible for the determination of an individual’s microbiome: diet and genetics. Although the diet seems to have a dominating effect and exerts this effect rapidly within days [57, 58], studies performed over the past decade underline the critical role of host genetics in shaping the metagenome, which we describe below. We then discuss examples from animal studies that illustrate mechanisms by which the immune system genome is involved in shaping the microbial metagenome.

### Effect of the host genome on the microbiome

A systematic study focusing on environmental factors and host genetic factors that together shape the complex microbial ecosystem showed that host genetics shape the diversity of the microbiome in mice [59]. In humans, the study of monozygotic twins sharing one genotype is a useful approach for evaluating a phenotype of interest. A study of monozygotic twins discovered that the microbiota is significantly more similar between twins than between unrelated individuals [60, 61]. Interestingly, host genetics and environmental factors may differentially influence distinct members of the microbiota, as the abundances of certain taxa, such as *Christensenellaceae*, is more highly correlated within monozygotic than within dizygotic twin pairs, whereas the abundance of other bacterial taxa, such as *Bacteroidetes*, seems to be mainly shaped by environmental factors [61, 62]. In addition to bacteria, monozygotic twins were shown to have more highly concordant gut archaea profiles, including those of the methanogen *Methanobrevibacter smithii*, than dizygotic twins [63].

On the basis of the association of certain host genomes with the abundance levels of microbial taxa [59], studies have been performed to identify regions of the genome that are responsible for these associations. A genetic linkage study examined a cross between the C57BL/6 J inbred mouse strain and an ICR/HaJ-derived outbred line to determine significant associations between host genomic variability and microbiota composition [59]. Eighteen quantitative trait loci (QTLs) of the host showed significant linkage with the relative abundances of specific microbial taxa. These host loci were shown to control individual microbial species, groups of related taxa, or groups of distantly related microorganisms and contained genes involved in immune signaling, such as *Irak3, Lyz1, Lyz2, Ifng*, and *Il22* [59]. Another genetic linkage study used a genetic resource based on a mouse inbred line, which is commonly used to study differences in susceptibility to obesity and other metabolic traits [64], to discover QTLs that influence gut microbial composition [65]. Additional genome sequencing in the QTL regions uncovered candidate genes that could account for the variation in microbiota composition. For example, a QTL located on chromosome 15, harboring the candidate gene *Irak4*, had a significant association with *Rikenellaceae*, whereas a QTL mapped on chromosome 12, harboring the candidate gene *Tgfb3*, had an effect on *Prevotellaceae*. Another QTL region on chromosome 4, located in a region rich in interferon genes, was associated with variation in the diversity and abundance of *Bacteroides* [65]. A 2015 study extended our insight into genomic control of the microbiome in humans by analyzing the microbial composition at 15 different body sites and their association to the host genome [66]. Interestingly, the strongest associations were driven by immune-pathway-related genes [66]. These studies strongly implicate the immune system as a major causative element in the determination of a host-specific microbiome and suggest that genomic variations in immune genes underlie, at least in part, inter-individual differences in microbiota composition.

Importantly, this inter-individual variability bears immediate relevance for inflammatory disease. For instance, abnormal interactions between the host and the microbiome are implicated in the pathogenesis of IBD. IBD is associated with substantial alterations in microbiome composition [67–69]. In the past few years, multiple host genetic loci associated with increased susceptibility to IBD were identified. To determine whether human genetic factors underlie the shifts in microbial populations that have been observed in a subset of patients with IBD, several studies have focused on the effect of known risk alleles for IBD on metagenomic stability. Of these, the innate immune receptor NOD2 and the autophagy-related protein ATG16L1 were significantly associated with shifts in microbial composition [70–73]. The relative abundances of several taxonomic groups, such as *Faecalibacterium* and *Escherichia*, were associated with NOD2 and ATG16L1 genotypes and disease phenotype. These results support the hypothesis that both genetic factors and disease manifestation drive substantial shifts in metagenomic composition.

### Mechanisms of microbiome regulation by the host immune system

What are the signaling pathways and effector mechanisms by which the host influences microbial colonization? In line with the genetic linkage studies discussed above, the antimicrobial activity of the immune system is a prime candidate for such activity, and mice with global deficiencies in the innate and adaptive arms of the immune system have been reported to harbor an altered microbiota composition [74]. The innate immune system is the first line of host defense against microbial pathogens. It functions through the sensing of microorganisms by innate immune receptors, which subsequently trigger an immune effector response. Innate immune sensing is also required for the activation of adaptive immunity, which is driven by antigen-specific T and B cells. In this section, we summarize some examples of findings that highlight the mechanisms by which the innate and adaptive immune responses shape the metagenome, then briefly discuss how diseases of the immune system affect the microbiome.

#### Innate immunity

Cells of the innate immune system express a wide array of signaling receptors that recognize signals from the microbiota as well as signals indicating tissue damage. Several studies using genetically modified mouse models have demonstrated the role of host innate immune components in influencing the composition of the gut microbiota, which modifies disease susceptibility [17]. Progress made in metagenomic sequencing has enabled the study of host innate immune control of microbial ecology. The findings indicate that several immune pathways contribute to maintaining a stable bacterial population, which potentially supports the existence of multiple levels of cross-talk, compensation, and complementarity in effector mechanisms of the innate immune system that exert control over the metagenome [75].

Toll-like receptors (TLRs) were the first pattern recognition receptors (PRRs) implicated in the recognition of ligands from commensal microbiota [76]. The TLR-induced inflammatory response is dependent on a common signaling pathway that is mediated by the adaptor molecule myeloid differentiation primary response gene 88 (MYD88) [77]. The co-receptor of TLR4, CD14, has been implicated in host–microbiota co-regulation in obesity [78]. Nevertheless, the role of TLR signaling in controlling the composition of the intestinal microbiota remains controversial. The intestinal microbiota of *Tlr5*-deficient mice and *Myd88*-deficient mice was described to differ from that of wild-type mice [79, 80]. However, another study showed that TLR signaling or MYD88 signaling does not change the composition of the intestinal microbiota under homeostatic conditions and after recovery from antibiotic treatment, but rather that parental lineage and housing conditions of mice are critical determinants of the microbiome [81]. This possibly implicates familial transmission as an additional strong driver of microbiome composition, which is in line with the interpretation of results from a study of human microbiome composition within and across households [2].

**Fig. 2:**
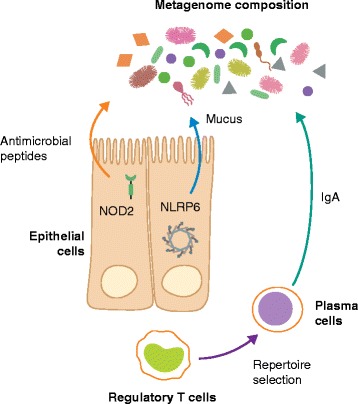
Effects of the innate and adaptive immune system on metagenome composition. Examples of how the innate and adaptive immune system controls microbiome composition via effector mechanisms. NOD2-dependent secretion of anti-microbial peptides is involved in shaping the configuration of the microbiota. NLRP6 controls mucus secretion by goblet cells, thus having an effect on the epithelial-proximal bacterial compartment. Regulatory T cells orchestrate B-cell-mediated IgA secretion. The IgA repertoire, in turn, controls bacterial targeting by the immune system and thus metagenomic composition. *IgA* immunoglobin A

Another family of pattern recognition receptors is the family of NOD-like receptors (NLRs), which sense a wide array of microbial ligands and host-derived signals of cell damage. NOD2 has an essential role in controlling the commensal bacterial community in the intestine (Fig. 2). Analysis of intestinal microbiota of *Nod2*-deficient mice revealed increased numbers of commensal bacteria, as well as a reduced capability to clear newly colonizing bacteria [82]. In addition to the control of the microbiota by NOD2, bacterial colonization was shown to induce the expression of NOD2. Mechanistically, NOD2 was suggested to have an important role in intestinal crypt function and regulate commensal microbiota composition and abundance through the bactericidal activity of antimicrobial peptides secreted by the ileal crypt. Consistently, several studies suggested that mutations in *Nod2* alter host–microbial interactions through altered antimicrobial activity [83, 84].

Certain NLR proteins are capable of forming a cytoplasmic complex called an inflammasome [85], including NLRP1, NLRP3, NLRC4, NLRP6, and NLRP7 [86]. Although these NLR proteins have different cell-type-specific expression patterns, all form an inflammasome complex that mediates the secretion of the cytokines IL-1β and IL-18, in a caspase-1 dependent manner [86]. Deficiency in the NLRP6 inflammasome, which is highly expressed in mouse colonic epithelial cells, results in reduced IL-18 levels and a dysbiotic microbiota characterized by expanded representation of the bacterial phyla *Bacteroidetes* (*Prevotellaceae*) and TM7 [87]. The development of dysbiosis in NLRP6-deficient mice resulted in exacerbation of chemical colitis induced by exposure to dextran sodium sulfate [87], colitis-associated colorectal cancer [88], and features of the metabolic syndrome, including non-alcoholic fatty liver disease, morbid obesity, and type II diabetes mellitus [89]. Co-housing of *Nlrp6*-deficient mice with wild-type mice resulted in dysbiotic microbial transfer to the recipient wild-type mice and exacerbation of colitis, cancer, hepatic steatosis, and obesity in the new host [87–89].

The NLRC4 inflammasome has been shown to be activated by Gram-negative bacteria [90, 91], enteric pathogens, including *Salmonella enterica* Typhimurium and *Shigella flexneri* [92, 93], and more specifically by bacterial virulence factors as well as bacterial flagellin [92]. NLRC4 was implicated in a specific innate immune response that can discriminate pathogenic from commensal bacteria in the intestine. In particular, NLRC4 recognizes invading pathogens in the gut, whereas commensal bacteria remain undetected [94].

An additional link between the host genotype and gut bacterial diversity was established when a mutation in the *MEFV* gene was shown to lead to changes in the human gut microbiota [95]. Mutations in *MEFV* cause the autoinflammatory disorder familial Mediterranean fever (FMF). The *MEFV* gene encodes the protein pyrin/marenostrin, which is involved in regulation of innate immunity [96] through suppression of IL-1β activation and abrogation of inflammation [97, 98]. Analysis of patients with FMF revealed the occurrence of significant changes in bacterial community structure during the active disease state [95]. These changes were characterized by reduced total numbers of bacteria, diminished bacterial diversity, and shifts in bacterial composition. In particular, the proportions of *Faecalibacterium* and *Parabacteroides* were substantially increased, whereas the proportion of *Prevotellaceae* was substantially lower in patients with acute disease than in control individuals [99]. However, the exact molecular mechanisms behind these alterations are not fully understood.

In addition to innate immune receptors, which take part in the communication between the host and its microbiota by sensing microbial presence, other antimicrobial substances can function as regulatory molecules that contribute to maintaining a stable microbial niche. Antimicrobial peptides are important effectors of innate immunity that seem to be involved in the maintenance of the symbiotic environment in the gut. The majority of antimicrobial peptides are cationic and amphipathic, which enables them to interfere with bacterial membrane stability. Defensins, which are secreted by Paneth cells of the small intestine, are particularly abundant and widely distributed antimicrobial peptides. The α-defensins were shown to contribute to host defense against enteric pathogens while shaping the composition of the small intestinal microbiota, as demonstrated in a mouse model of defensin overexpression and defensin deficiency [100, 101]. Moreover, RegIIIγ, a secreted antibacterial lectin that specifically targets Gram-positive bacteria, was shown to have a role in maintaining host-microbial homeostasis by keeping the bacteria separated from the epithelium. In RegIIIγ-deficient mice, more bacteria reach the small intestinal epithelium and trigger an intestinal adaptive immune response than in control mice [102].

#### Adaptive immunity

In addition to the innate immune system, cells of the adaptive immune system have been reported to participate in shaping the microbiome structure. This influence was highlighted by the consequences of the absence of adaptive immune system cells in Rag1-deficient mice, which have an aberrant microbial community [103]. Kawamoto et al. narrowed down the cell types responsible for these aberrances in Rag-deficient mice by using wild-type mice lacking T cells, which were found to have reduced microbial diversity in the intestine [104]. Administration of *Foxp3*^+^ regulatory T cells to T-cell-deficient mice restored bacterial diversity. This effect was dependent on the presence of class-switched antibodies and immunoglobulin A (IgA) selection (Fig. 2). Therefore, *Foxp3*^+^ regulatory T cells were suggested to mediate the targeting of commensal bacteria by IgA to maintain a homeostatic bacterial diversity [104]. Indeed, several studies have shown that affinity maturation, class-switch recombination and somatic hypermutation of IgAs have a crucial role in the regulation of bacterial composition in the intestine [104–107].

The mechanism that controls antibody diversity is tightly regulated by activation-induced deaminase (AID). AID produces diversity by converting cytosine to uracil within the immunoglobulin loci. Deficiency in AID was shown to result in the expansion of anaerobic bacteria in the small intestine [105], which was later confirmed when Aid-deficient mice were analyzed using a sequencing-based method and observed to have predominant expansion of segmented filamentous bacteria throughout the small intestine [108]. An additional study focused on the role of somatic hypermutation in mucosal immunity by analyzing mice with a point mutation in the Aicda gene [107]. The mutated mice were shown to produce normal amounts of unmutated IgA and immunoglobulin M (IgM), which cannot efficiently recognize the intestinal microbiota, thereby causing aberrant expansion and epithelial adhesion of certain bacterial species. These findings further corroborate the importance of AID and antibody diversity in the maintenance of a homeostatic microbiome (Fig. 2). Mechanistically, the selection of the plasma cell IgA repertoire that targets the intestinal microbiota requires the inhibitory co-receptor programmed cell death 1 (PD-1). PD-1 controls the number and phenotype of T follicular helper cells in the germinal centers of Peyer’s patches, which in turn control the selection of IgAs and microbial communities in the gut [109].

#### Diseases of the immune system

Given the wealth of evidence for an involvement of the immune system in shaping the microbiome during homeostasis, it is interesting to consider whether diseases of the immune system cause aberrations in the composition and function of the microbiome. In cases of severe immunomodulation by inflammatory disease, iatrogenic intervention, or immunodeficiency, one might find consequent changes in the microbiota. These, in turn, may reciprocally influence the disease process. Indeed, many such examples have been found, including graft-versus-host disease [110], kidney transplantation [111], hepatitis [112], cirrhosis [113], psoriasis [114], IBD [115], and arthritis [116]. Furthermore, immunosuppressive drugs affect the balance between microbiota and the immune system [117]. The effect of the immune system on the composition of the microbiome becomes apparent when considering together various human conditions of immune system dysfunction.

## Conclusions and future directions

The intersection between metagenomics and immunogenomics, which has only recently begun to be explored, has fundamental importance for human health. Although the phenomenology of the cross-regulation of gene expression between commensal bacteria and cells of the immune system has been established in the past few years, our mechanistic understanding of this relationship is still in its infancy [118]. It is now firmly established that commensal microorganisms influence host gene expression, in the gastrointestinal tract and in other systems, and that both microbial cell components and secreted metabolites are involved in the transcriptional response of the host to microbial colonization. Nonetheless, some major questions remain. What are the mechanisms by which microbiota-derived molecules are integrated into the transcriptional circuits of host cells? How is the cell-type-specificity of transcriptional responses to the microbiota ensured? How does the biogeographical localization of commensal species at different mucosal surfaces influence organ-specific gene regulation? What are the kinetics of transcriptional control by the microbiota? Answering these questions would enable the field to enter a stage in which interventional strategies could be designed, with the goal of actively controlling host transcription through harnessing the pathways used and the molecules secreted by commensal microorganisms to influence host gene regulation. Such strategies could help investigate the use of dietary modulation approaches to modify the levels of microbial metabolites upstream of gene regulatory circuits.

As outlined in this review, major signaling pathways of the innate immune system are involved in shaping the host-microbiota cross-talk during homeostasis. Interestingly, genes that are part of these very pathways are among the most frequently affected genes in common inflammatory disorders, such as IBD [119], which strongly suggests that a disturbed host–microbiota interaction is fundamentally involved in the etiology of these diseases [115, 120]. Of note, IBD-associated microbiomes have consistently been reported to have low bacterial diversity [121], which implies that a high bacterial diversity is a favorable condition during homeostasis and disease.

Furthermore, although it has been established that the host genome shapes the composition of the microbial metagenome, our knowledge of the evolutionary benefits of the microbial-driven regulation of host gene expression remains sparse. Is adaptation of host gene expression to the microbial status necessary for the homeostatic maintenance of commensal communities? Which parameters characterize a 'desirable' microbial metagenome and are selected by the host immune system? The genomic revolution has opened the door for such questions to be explored, and the next decade in the study of genomic interactions between the immune system and the microbiome will certainly transform our understanding of the eukaryotic–prokaryotic cross-talk that characterizes the mammalian 'super-organism'.

## Abbreviations

AID: activation-induced deaminase
FMF: familial Mediterranean fever
HDAC: histone deacetylase
IBD: inflammatory bowel disease
IgA: immunoglobulin A
IL: interleukin
ILC: innate lymphoid cell
NKT: natural killer T
NLR: NOD-like receptor
PD-1: programmed cell death 1
PRR: pattern recognition receptor
QTL: quantitative trait locus
T_h_17: T helper 17
TLR: Toll-like receptor
